# Changes in Running Economy During a 65-km Ultramarathon

**DOI:** 10.3389/fphys.2018.01809

**Published:** 2018-12-13

**Authors:** Volker Scheer, Solveig Vieluf, Leoni Cramer, Rasmus Jakobsmeyer, Hans-Christian Heitkamp

**Affiliations:** ^1^Ultra Sports Science Foundation, Pierre-Bénite, France; ^2^Department of Exercise and Health, Institute of Sports Medicine, Paderborn University, Paderborn, Germany

**Keywords:** oxygen consumption, physiology, endurance, energy cost of running, oxygen cost of running

## Abstract

**Purpose:** Running economy (RE), expressed as oxygen cost (O_2_ cost) and energy cost of running (Cr) is important in ultramarathon (UM) running as it can help predict race performance. Controversy remains if RE increases, decreases, or remains stable in UM running. We examined RE before, during, and after a 65-km UM.

**Methods:** 15 male UM runners (mean age 45 ± 5.7 years) completed a standard exercise test (mean VO_2_max 48.8 ± 3.4 ml⋅kg^-1^⋅min^-1^) for determination of the individual testing speed (60% VO_2_max: mean speed 9.4 ± 0.7 km/h). This was followed by a 65-km UM (elevation ± 1093 m) consisting of three laps (each 21.7 km). Pre and post indirect calorimetry measurements at individual running speed on the treadmill at UM-specific slopes (average percentage of positive and negative elevation) at -3, +3%, and level grade were performed in randomized order on a motorized treadmill in the laboratory for calculation of RE. Additionally after each lap, testing at +3% took place.

**Results:** The O_2_ cost, Cr, and RER increased significantly pre to post UM (*p* < 0.01). During the uphill running, a main effect of distance indicated a gradual, linear increase in O_2_ cost, *F*(2,28) = 5.81, *p* < 0.01, $\eta_{p}^{2}$ = 0.29, and Cr, *F*(2,28) = 5.96, *p* = 0.01, $\eta_{p}^{2}$ = 0.30.

**Conclusion:** O_2_ cost and Cr increased significantly pre to post UM in all testing conditions as well as during the uphill testing throughout the UM. This is the first study to demonstrate a consistent increase in O_2_ cost and Cr among a range of different slopes, at individual running speeds and race-specific slopes giving further evidence that these measures of RE increase in UM running.

## Introduction

Popularity in ultramarathon (UM) running has increased over the years and includes race distances in excess of the traditional marathon distance of 42.195 km (Scheer, 2018). They are often held in challenging environmental conditions, testing the limits of human endurance (Scheer et al., 2015). Considerable research has focused on the medical and physiological aspects of UMs (Millet et al., 2000, 2011a; Scheer and Murray, 2011; Millet and Millet, 2012; Costa et al., 2013; Hoffman et al., 2014; Scheer et al., 2014; Vernillo et al., 2017a), but up to now, controversy remains on the role of running economy (RE) in UM running (Vernillo et al., 2017b). RE is important as it is classically used in conjunction with VO_2_max and percentage of VO_2_max to predict race performance (Barnes and Kilding, 2015; Scheer et al., 2018a). RE is a multifactorial concept that combines the function of the metabolic, cardiopulmonary, biomechanical, and neuromuscular system (Barnes and Kilding, 2015) and can either be expressed as oxygen consumption (in ml⋅kg^-1^⋅min^-1^), oxygen consumption to cover a given distance [oxygen cost (O_2_ cost), e.g., in ml O_2_⋅kg^-1^⋅km^-1^] or as the energy cost of running (Cr) expressed in units of energy (J⋅kg^-1^⋅m^-1^) (Fletcher et al., 2009; Barnes and Kilding, 2015; Vernillo et al., 2015, 2017a). Cr is of particular interest in UM as it reflects the energy demand in prolonged exercise where a shift from carbohydrate to fat substrate utilization can be observed (Schena et al., 2014; Vernillo et al., 2015, 2017a). Cr typically increases up to marathon distances (Brueckner et al., 1991), but in UM, there is debate if Cr really increases after an UM (Vernillo et al., 2017b) as some studies suggest (Gimenez et al., 2013; Vernillo et al., 2015), remains stable (Schena et al., 2014; Vernillo et al., 2014, 2015; Balducci et al., 2017; Savoldelli et al., 2017), or decrease post UM (Vernillo et al., 2014, 2016). Data pertaining the O_2_ cost of running are more uniform, with the majority of studies demonstrating an increase (Lazzer et al., 2012, 2015; Schena et al., 2014) or no change post UM (Millet et al., 2000, 2009; Fusi et al., 2008; Lazzer et al., 2014; Balducci et al., 2017). Most studies have investigated RE changes pre to post UM (Vernillo et al., 2014, 2016; Lazzer et al., 2015; Balducci et al., 2017) but only one study to date (Schena et al., 2014) investigated RE during a 60-km UM in field conditions that found no significant changes in Cr. Some of those discrepancies observed may be due to methodological differences in study designs (Vernillo et al., 2017b). It has recently been suggested that future studies should take into consideration the specific UM race characteristics and profile and use individual testing speeds and race-specific gradients for RE testing on the treadmill after sufficient familiarization with testing conditions (Vernillo et al., 2017b).

The purpose of our study was to investigate RE (Cr and O_2_ cost) before, during, and after a 65-km UM, addressing some of those methodological concerns raised and examining runners at individual running speeds and different specific gradient slopes (level, uphill, and downhill) on the treadmill according to the specific UM profile. Our hypothesis was that Cr and O_2_ cost would increase with increasing running distance and among different gradients.

## Materials and Methods

### Participants

Fifteen experienced male UM runners (age 44.9 ± 5.7 years, height 178.3 ± 4.9 cm, weight 78.4 ± 8.4 kg, BMI 24.6 ± 2.2 kg/m^2^, running experience 12.2 ± 8.6 years, previous UM experience 5 ± 4.2 races, training kilometers per week 75 ± 19.3 km) volunteered to participate in the study. Only healthy male athletes, between the ages of 18 and 60 years, who previously participated in at least one UM of more than 60 km, were allowed to participate. Runners who had sustained a recent injury (less than 3 months before the event) were excluded. They were recruited through announcements at local running clubs and local Internet groups for runners. All participants were informed of the protocol prior to the first test and provided written, informed consent. The internal review board of the local medical council (Ärztekammer Westfalen-Lippe) and the University of Münster, Germany, approved all procedures (approval number 2017-465-f-S), and research was conducted in accordance with the Declaration of Helsinki.

### Study Design

The study design consisted of two test days. Participants were instructed to abstain from consuming alcohol or caffeine and refrain from strenuous and exhaustive exercise 24 h prior to each test day. On test day 1, participants received a medical checkup, consisting of medical history and examination of the musculoskeletal and cardiovascular system, resting blood pressure (Adult 11, durable blood pressure cuff, Welch Allyn, Skaneateles, NY, United States) and resting ECG (Cardio 100 BT, Custo, Ottobrunn, Germany) by the attending physician to exclude any significant underlying pathologies. All participants were attested to be in good health and able to participate in the study. Participants were familiar with treadmill running and were further familiarized with the specific running protocol on the treadmill (h/p/cosmos Pulsar 3p; Traunstein, Germany). This was followed by a standard graded exercise test (starting at 6.0 km/h, step duration 3 min, increased by 2.0 km/h, and inclination 1.0%) until task failure with continuous breath by breath measurements for ventilatory parameters (Metalyzer 3B, Cortex Biophysik, Leipzig, Germany) (Scheer et al., 2018b). To determine individual running speed for treadmill tests on test day 2, the speed at 60% VO_2_max was extrapolated from the VO_2_–work rate relationship. This intensity was selected as it corresponds to intensities observed during 6-h events (Davies and Thompson, 1979).

### Ultramarathon

Test day 2 included four running tests on the treadmill, two venous blood samples, and a 65-km UM. Test day 2 was conducted on three separate occasions due to limitations of equipment and staff capacity, to ensure smooth testing procedures with little to no waiting times (maximum number of six participants per day). For the same reason, RE measurements during the UM could only be conducted at +3% incline. Tests took place in January and February 2018 with comparable outdoor conditions (average temperature + 6°C) for the UM. The UM was designed as a loop course (a total of three laps) starting and finishing at our university department. Each lap measured 21.7 km with a cumulative ascent of 364.4 m and cumulative descent of 364.4 m (Garmin Forerunner 735XT), equating to a total distance of 65 km (±1093 m). The terrain consisted predominately of forest tracks, gravel, and tarmac paths. It contained several uphill and downhill running sections and the average slope measured 3% (positive and negative elevation), which was used as the incline/decline for further tests (uphill/downhill) on the treadmill as outlined below. Participants were allowed to select their own individual running speed during the UM and were allowed to drink and eat *ad libitum*. During the course, runners were self-sufficient but after each lab food and drink was provided to them at the university.

### Running Economy

Treadmill tests (A–D) were standardized and included continuous measurements of ventilatory parameters including oxygen uptake ($\dot{V}$), carbon dioxide output ($\dot{V}$CO_2_), and respiratory exchange ratio (RER) after calibration before each test (Scheer et al., 2018b). Heart rate values were obtained *via* Bluetooth (Polar T31 sensor) and participants were asked to provide rate of perceived exertion (RPE) on the 6- to 20-point Borg scale immediately prior and at the end of each test (Borg, 1985). Test A (just before the start of the UM) and test D (immediately after the finish of the UM) were conducted in the same fashion and consisted of three 5-min running intervals on the treadmill at 60% VO_2_max of the individual running speed in randomized order at level grade, +3% incline, and -3% decline, with a one minute rest period between intervals. Test B (after one lap of running) and Test C (after two laps of running) where conducted in the same fashion but only at +3% incline of the treadmill. To calculate RE, $\dot{V}$O2, $\dot{V}$CO_2_, and RER were obtained from the last minute of treadmill running during steady state conditions and were filtered into 5-s blocks for data analyses as described by Vernillo et al. (2015). Net VO_2_ values were obtained from calculating the difference of VO_2_ at steady state minus $\dot{V}$O_2_ at rest and for determination of the caloric equivalent of $\dot{V}$O_2_ values were converted depending on RER values (Péronnet and Massicotte, 1991). RE was expressed as both net O_2_ cost of running (O_2_⋅kg^-1^⋅km^-1^) and net Cr of running (Cr in J⋅kg^-1^⋅m^-1^) (Vernillo et al., 2015).

### Blood Sampling

Venous blood samples were obtained immediately prior and post UM in resting supine position from the antecubital vein by the attending physician. Blood parameters obtained included creatine kinase (CK), C-reactive protein (CRP), and leucocyte count.

### Statistical Analyses

Statistical analyses were conducted in SPSS for Windows version 20.0 (IBM Corp., Armonk, NY, United States). The relative change [relative change = (post - pre)/pre × 100] was expressed as the difference between pre and post measure in relation to pretest values in per cent and was calculated for RER, O_2_ cost and Cr. Rate of change was tested with one-sample tests. Standard significance level was set to *p* < 0.05 and corrected for multiple comparisons by Bonferroni correction (α′= 0.05/5 = 0.01). To test for differences between tests, repeated measure ANOVA with three tests (downhill, level, and uphill) was calculated for the relative change of RER, O_2_ cost, and Cr. To analyze changes continuously during the uphill test, RER, O_2_ cost, and Cr changes in relation to pretest measures were analyzed with a repeated measure ANOVA with the factor three distance (21.7, 43.4, and 65 km). Greenhouse–Geisser adjustment was reported in case the sphericity assumption was violated. Effect sizes were reported as partial eta squares ($\eta_{p}^{2}$). *Post hoc* tests were corrected with Bonferroni. *p*-Values were corrected by SPSS. Based on violation of normal distribution, we calculated Spearman correlations between the relative changes of RER, O_2_ cost, Cr and the blood parameters as well as the numerical values of the RPE.

## Results

**FIGURE 1:**
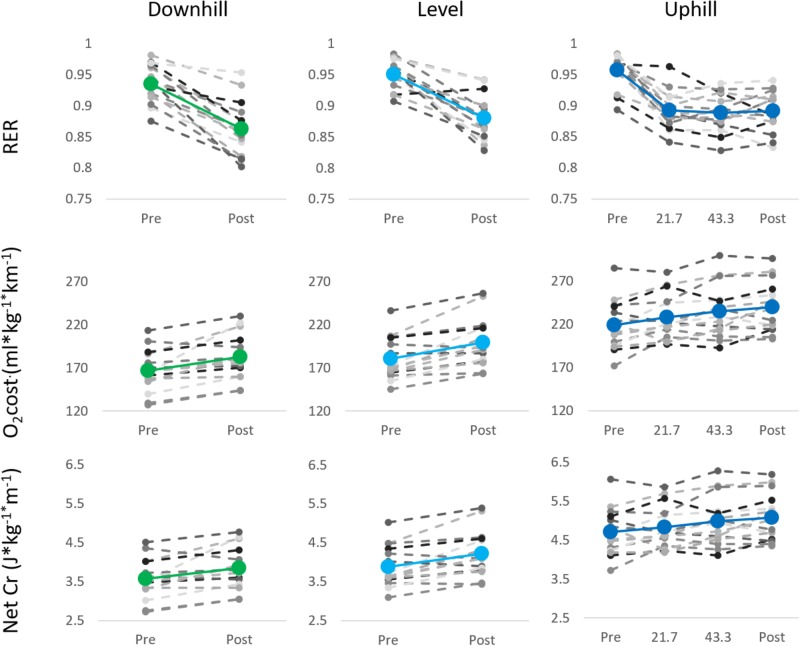
Mean values of RER, O_2_ cost, and Cr (colored marker) and individual for each UM runner (gray markers) during the different tests.

The values from the initial exercise test, pre and post UM blood parameters, and values of the RPE with their relative change and correlation to O_2_ cost and Cr are shown in Table 1. All blood markers and the RPE increased significantly pre to post UM, but their relative changes did neither correlate with O_2_ cost nor Cr apart from RPE values. Absolute mean values (±SD) were the following for RER, O_2_ cost, and Cr pre vs. post for level (0.95 ± 0.02 vs. 0.88 ± 0.04, 180.60 ± 24.75 vs. 199.47 ± 28.43 O_2_⋅kg^-1^⋅km^-1^, and 3.88 ± 0.52 vs. 4.22 ± 0.59 J⋅kg^-1^⋅m^-1^) and downhill running (0.94 ± 0.03 vs. 0.86 ± 0.04, 166.60 ± 24.25 vs. 182.40 ± 26.58 O_2_⋅kg^-1^⋅km^-1^, and 3.57 ± 0.52 vs. 3.85 ± 0.55 J⋅kg^-1^⋅m^-1^). During the four different time points in uphill running RER, O_2_ cost, and Cr were as follows: 0.96 ± 0.03 vs. 0.89 ± 0.03 vs. 0.89 ± 0.03 vs. 0.89 ± 0.03, 218.87 ± 28.29 vs. 227.93 ± 30.45 vs. 234.93 ± 30.63 vs. 239.93 ± 29.17 O_2_⋅kg^-1^⋅km^-1^, and 4.71 ± 0.59 vs. 4.83 ± 0.54 vs. 4.98 ± 0.63 vs. 5.08 ± 0.60 J⋅kg^-1^⋅m^-1^. Graphical presentation of the mean values and data of each individual runner for RER, O_2_ cost, and Cr for downhill, level, and uphill running at the different time points can be seen in Figure 1. Calculated relative changes are presented in Table 2. Relative changes for RER showed changes through all pre to post-tests during level and downhill running and all subsequent measures during uphill running. This was also observed with O_2_ cost and Cr during level and downhill running and after 21.7 km and subsequent tests in uphill running (see Table 2). All protocols showed significant relative changes from pre to post test, as indicated by significant one sample *t*-test for the parameters RER, O_2_, and Cr (see Table 2). Comparing the relative changes across protocols, repeated measure ANOVA revealed no main effect of protocol for RER, *F*(2,28) = 0.55, *p* = 0.58, $\eta_{p}^{2}$ = 0.04, O_2_ cost, *F*(2,28) = 0.11, *p* = 0.83, $\eta_{p}^{2}$ = 0.01, and Cr, *F*(2,28) = 0.08, *p* = 0.86, $\eta_{p}^{2}$ = 0.01. In addition to pre and post measures, the uphill test was conducted after 21.7 and 43.4 km, to illustrate the progression of change. Analysis revealed no main effect of distance for RER, *F*(2,28) = 0.20, *p* = 0.82, $\eta_{p}^{2}$ = 0.01. For both measures of RE, O_2_ cost, *F*(2,28) = 5.81, *p* < 0.01, $\eta_{p}^{2}$ = 0.29, and Cr, *F*(2,28) = 5.96, *p* = 0.01, $\eta_{p}^{2}$ = 0.30, a significant main effect of distance was revealed. Bonferroni corrected *post hoc* comparisons showed O_2_ cost (*p* = 0.04) and Cr (*p* = 0.03) increased more at post-test than after 21.7 km.

**Table 1:** Descriptive results [mean values and standard deviation (SD)] from the initial exercise test, blood parameters and RPE pre and post UM, with relative change and correlation to O_2_ cost and Cr.

		Pre	Post	Relative change		Correlation with O_2_ cost	Correlation with Cr
VO_2_max (ml⋅km^-1^⋅min^-1^)	Mean (SD)	48.80 (3.41)					
vVO_2_max (km/h)	Mean (SD)	16.22 (1.00)					
60% vVO_2_max (km/h)	Mean (SD)	9.42 (0.69)					
Leukocytes (10^9^/L)	Mean (SD)	5.58 (1.62)	14.75 (3.20)	174.84^*^ (68.32)	*r* (*p*)	-0.37 (0.17)	-0.38 (0.17)
CK (U/L)	Mean (SD)	172.27 (71.79)	681.53 (393.64)	311.29^*^ (178.09)	*r* (*p*)	0.31 (0.27)	0.29 (0.29)
CRP (mg/L)	Mean (SD)	0.09 (0.06)	0.17 (0.13)	113.49 (98.61)	*r* (*p*)	0.31 (0.26)	0.30 (0.27)
RPE (Borg)	Mean (SD)	11.27 (1.75)	15.93 (1.83)	44.18^*^ (24.27)	*r* (*p*)	-0.45 (0.09)	-0.47 (0.08)


*^∗^Indicates that one-sample tests reached significance with Bonferroni correction.*

**Table 2:** Mean values and standard deviations (SD) as well as results of one-sample tests (*df* = 14 and ^∗^ indicates significance after Bonferroni correction) relative changes per group for all measures in the three different tests and distances.

		RER	O_2_ cost	Cr
**Level**				
Post UM	Mean (SD)	-7.40 (3.88)	10.66 (8.33)	8.98 (8.04)
	T; *p*	-7.37; <0.01^∗^	4.96; <0.01^∗^	4.33; <0.01^∗^
**Downhill**				
Post UM	Mean (SD)	-7.74 (3.55)	9.86 (9.39)	8.34 (9.47)
	*T*; *p*	-8.44; <0.01^∗^	4.07; <0.01^∗^	3.41; <0.01^∗^
**Uphill**				
21.7 km	Mean (SD)	-6.81 (2.81)	4.56 (7.40)	2.90 (7.47)
	*T*; *p*	-9.37; <0.01^∗^	2.39; 0.03	1.50; 0.15
43.4 km	Mean (SD)	-7.19 (2.37)	7.58 (7.40)	5.98 (7.28)
	*T*; *p*	-11.77; <0.01^∗^	3.97; <0.01^∗^	3.18; <0.01^∗^
Post UM	Mean (SD)	-6.91 (2.86)	9.97 (7.45)	8.34 (7.53)
	T; *p*	-9.35; <0.01^∗^	5.19; <0.01^∗^	4.29; <0.01^∗^


## Discussion

Our main findings are that Cr and O_2_ cost increased significantly pre to post UM in all testing conditions as well as during the uphill testing throughout the UM. This is in line with our hypothesis demonstrating a consistent increase in these measures of RE.

### O_2_ Cost of Running

Oxygen cost of running increased by about 10% for uphill and downhill and about 11% for level grade running conditions pre to post UM. This is in line with most previous studies that showed an increase in the O_2_ cost of running post UM (Lazzer et al., 2012, 2015; Schena et al., 2014). A new finding is that during the UM in the uphill running conditions a significant gradual linear increase was observed at the different time points.

### RER and Energy Cost of Running

However, it is also known that the O_2_ cost of running, especially in UM running can be overestimated and an expression of RE as Cr is a much better way of describing RE as it takes into account the caloric equivalent of VO_2_ (Péronnet and Massicotte, 1991; Vernillo et al., 2017b). RER values are therefore important, and it is known that they decrease over time with exercise especially post UM (Schena et al., 2014; Vernillo et al., 2015), in line with our own result. In prolonged exercise, a progressive depletion of glycogen stores occur with a shift in substrate utilization from carbohydrates to fat resulting in decreased RER values (Schena et al., 2014). Interestingly, that decrease was already observed after 21.7 km, suggesting that the shift in substrate utilization had occurred at or before this time point.

Changes in RER values will have an obvious impact in calculating Cr when for the determination of the caloric equivalent $\dot{V}$O_2_ values are converted depending on RER values (Péronnet and Massicotte, 1991). Cr has been shown to increase in running distances up to marathon (Brueckner et al., 1991), but in UM, there is still some debate if Cr increases (Gimenez et al., 2013; Vernillo et al., 2015), decrease (Vernillo et al., 2014, 2016) or remains stable after an UM (Fusi et al., 2008; Schena et al., 2014; Vernillo et al., 2014, 2015; Balducci et al., 2017; Savoldelli et al., 2017). Our results showed a significant increase in Cr across all testing conditions in downhill, level, and uphill running pre to post UM of between 8 and 9%. Vernillo et al. (2015) reported a 13.1% increase in post-race Cr in downhill running conditions only with no differences in level or uphill Cr post UM over a similar distance of 65 km but with a much steeper elevation of +4000 m and fixed treadmill testing conditions for participants regarding slope and speed (±5% and 10 km/h) (Vernillo et al., 2015). A new finding is that we also observed a significant gradual increase of Cr during uphill running conditions throughout the UM. To our knowledge, there is only one other study to date examining Cr changes during an UM in field conditions (Schena et al., 2014). A non-significant, but small gradual increase of Cr was observed, during a flat 60-km UM course, with Cr measured at level grade running on a 400-m running track at self-selected speed equating to speeds of 65–70% VO_2_max (Schena et al., 2014). Another study (Gimenez et al., 2013) examined RE during a 24-h treadmill run, demonstrating an increase in oxygen consumption and concomitant decrease of RER in the first 8 h, thus remaining stable thereafter. This resulted in an increased Cr during that time period (Gimenez et al., 2013) The mechanisms behind the increase in Cr after an UM are not fully understood but changes in the neuromuscular system and biomechanical factors seem to play an important role (Millet et al., 2011b; Degache et al., 2013; Vernillo et al., 2017b).

### Neuromuscular Factors and Fatigue

Muscle fatigue and skeletal muscle damage can lead to changes in neural input to compensate decreasing muscle force especially during push off phase of the gait pattern (Millet et al., 2011b; Vernillo et al., 2017b). Biomechanical changes can alter gait pattern and increased stride frequency, and leg and tendon stiffness have been observed leading to compensatory adjustments in the gait pattern through the gait cycle (Millet et al., 2011b; Degache et al., 2013; Vernillo et al., 2015, 2017b). These changes require an increasing VO_2_ demand leading to increases in Cr. For those studies that have shown an improvement in Cr post UM (Vernillo et al., 2014, 2016) the underlying mechanism are not clear either. Positive neuromuscular adaptations and control of fatigued muscles may lead to improvements in movement through redistribution from motor units of fatigued to non-fatigued muscle groups (Giandolini et al., 2016; Vernillo et al., 2017b). This may lead to a preservation of a more economical running pattern (Schena et al., 2014) which may be of importance in extreme UM that have been investigated over a distance of 330 km and +24.000 m elevation (Vernillo et al., 2016) but this is not comparable to our current UM run.

Muscle enzyme (CK) and inflammatory markers (CRP and leukocytes) showed a significant increase from pre to post UM as described in previous studies (Schena et al., 2014; Gill et al., 2015). An increase of Cr with increasing muscle damage and raised CK have been observed (Kyröläinen et al., 2000); however, the correlation between muscle damage and changes in Cr are unclear (Schena et al., 2014) In our group, this increase was not correlated to and could not explain the rising O_2_ cost or Cr, suggesting other mechanisms to be involved. This could be further substantiated by the increase of the RPE that correlated with the rising O_2_ cost and Cr, demonstrating that fatigue or the subjective rating of effort and fatigue may play a role as previously suggested (Millet et al., 2011b; Gimenez et al., 2013).

### Methodological Considerations

Methodological concerns may be an explanation of the discrepancies observed in Cr in the different studies. Race distances, race profiles, and race conditions have varied widely with races ranging from 43 to 330 km and 24,000 m elevations, to single case reports of a 8500-km expedition, making comparisons difficult (Millet et al., 2009; Lazzer et al., 2015; Vernillo et al., 2016, 2017a). Running speed and intensity have also varied widely ranging from speeds at 40–80% of VO_2_max (Lazzer et al., 2012; Vernillo et al., 2017b). Vernillo et al. (2017b) had therefore suggested to include specific testing conditions of the UM, assessing the consistency of the individual responses, providing adequate familiarizations sessions to participants to the specific protocol, and inserting a control group for future studies.

### Strength

The main strength of the study is that we addressed some of the methodological considerations raised in the literature (Vernillo et al., 2017b). We included a preceding exercise test, to determine individual running speeds and participants received an adequate familiarization session of the testing protocol. The test protocol was designed according to specific slope conditions of the actual UM topography and tests were conducted not only pre- and post-race but also during the UM. Individual responses are presented in the results. However, we recognize that there are still some limitations and weaknesses to our study.

### Limitations

Testing took place on a treadmill and outdoor running condition and surface differ; however recently, it has been shown that although subtle differences exist between overground and treadmill running, overground running can reasonably be replicated on the treadmill (Firminger et al., 2018). Outdoor testing in the field can be done with a mobile spirometry unit as has been demonstrated (Savoldelli et al., 2017); however, this may lead to other biases as for example testing speed and ambient conditions cannot be controlled as in laboratory conditions.

Other factors such as changes in running biomechanics can affect RE; however, we were not able to conduct kinematic analyses in this current study. Testing took place over three test days due to limitations in laboratory and staff capacity; however, ambient conditions at the three test days were comparable so this should not have had an impact on the results. The study was conducted as a simulated 65 km run and not under race conditions, so some participants may not have reached their full potential, but considering the distance covered and the time needed to complete that distance this should not have negatively impacted the results obtained.

### Future Considerations

Examining changes in RE in the field, during different time points at varying UM distances and gradient slopes will be interesting, especially in groups of homogenous UM runners or elite UM runners. The insertion of a control group (Vernillo et al., 2017b) may also yield new insights.

## Conclusion

Energy cost of running and O_2_ cost increased significantly pre to post UM in all testing conditions as well as during the uphill testing throughout the UM. This is the first study to demonstrate a consistent increase in these measures of RE among a range of different slopes, at individual running speeds and race-specific slopes giving further evidence that Cr and O_2_ cost indeed increase in UM running.

## Author Contributions

VS and H-CH contributed substantially to the conception and design of this study. VS and LC contributed to data collection. SV carried out the data analysis and interpretation together with VS, LC, RJ, and H-CH. VS wrote the first draft of the manuscript, and all authors were involved in revising it critically. All authors gave final approval of the version to be published and agreed to be accountable for all aspects of this work.

## Conflict of Interest Statement

The authors declare that the research was conducted in the absence of any commercial or financial relationships that could be construed as a potential conflict of interest.
